# cAMP-PKA-CaMKII signaling pathway is involved in aggravated cardiotoxicity during Fuzi and Beimu Combination Treatment of Experimental Pulmonary Hypertension

**DOI:** 10.1038/srep34903

**Published:** 2016-10-14

**Authors:** Pengwei Zhuang, Yingying Huang, Zhiqiang Lu, Zhen Yang, Liman Xu, Fengjiao Sun, Yanjun Zhang, Jinao Duan

**Affiliations:** 1 Chinese Materia Medica College, Tianjin University of Traditional Chinese Medicine, Tianjin, China; 2 Tianjin State Key Laboratory of Modern Chinese Medicine, Tianjin University of Traditional Chinese Medicine, Tianjin, China; 3 School of Pharmaceutical Science and Technology, Tianjin University, Tianjin 300072, China; 4 Jiangsu Key Laboratory for High Technology Research of TCM Formulae, Nanjing University of Chinese Medicine, Nanjing, China

## Abstract

*Aconiti Lateralis Radix Praeparata* (Fuzi) and *Fritillariae Thunbergii bulbus* (Beimu) have been widely used clinically to treat cardiopulmonary related diseases in China. However, according to the classic rules of traditional Chinese medicine, Fuzi and Beimu should be prohibited to use as a combination for their incompatibility. Therefore, it is critical to elucidate the paradox on the use of Fuzi and Beimu combination therapy. Monocrotaline-induced pulmonary hypertension rats were treated with either Fuzi, Beimu, or their combination at different stages of PH. We demonstrated that at the early stage of PH, Fuzi and Beimu combination significantly improved lung function and reduced pulmonary histopathology. However, as the disease progressed, when Fuzi and Beimu combination were used at the late stage of PH, right ventricular chamber dilation was histologically apparent and myocardial apoptosis was significantly increased compared with each drug alone. Western-blotting results indicated that the main chemical ingredient of Beimu could down-regulate the protein phosphorylation levels of Akt and PDE4D, whereas the combination of Fuzi and Beimu could up-regulate PKA and CaMKII signaling pathways. Therefore, we concluded that Fuzi and Beimu combination potentially aggravated the heart injury due to the inhibition of PDK1/Akt/PDE4D axis and subsequent synergistic activation of βAR-Gs-PKA/CaMKII signaling pathway.

Pulmonary hypertension (PH) is a progressive and fatal hemodynamic problem, characterized by sustained raises in pulmonary arterial pressure and pulmonary vascular resistance, as well as thickening of small pulmonary arteries^1^. Right ventricular dysfunction has been considered as the crucial factor for deaths in patients with PH^2^,^3^. However, the mechanisms about how the alterations in right ventricular lead to heart failure remain insufficient^4^,^5^. Complementary and alternative medical products are usually used to treat PH in clinic (e.g. Beimu, Banxia, Gualou and Fuzi in Chinese), and some of these effective herbs were usually used as combinations to achieve a better efficacy.

Drug combination shows a unique advantage in the treatment of chronic and complex disease (e.g. COPD), however, the safety of combination therapy should not be ignored. Thousands-years clinical practice in traditional Chinese medicines (TCMs) has led to a series of theories and principles to guide the appropriate use of TCMs. Among them, “Eighteen antagonisms” is one of the fundamental principles on TCM incompatibility^6^. Two TCMs, namely *Aconiti Lateralis Radix Praeparata* (Fuzi) and *Fritillariae Thunbergii bulbus* (Beimu), are widely used in clinic to treat various heart and pulmonary diseases in China^7^,^8^,^9^,^10^. In some cases, Fuzi and Beimu have been used as a combination therapy to achieve a better clinical performance, such as corpulmonale and asthma^11^,^12^, which is contrary to the rule of “Eighteen antagonisms” for potential fatal consequences resulted from the combination use of Fuzi and Beimu. Therefore, the clinical disease and symptom conditions were thought to be the key terms leading to toxicity or efficacy of the co-use of Fuzi and Beimu. However, whether and how Fuzi and Beimu combination leads to the fatal incompatibility remains unknown.

Sympathetic adrenergic activity is highly elevated in PH^13^. Sympathetic over-activity is a compensatory mechanism to maintain cardiac function at first, but might be detrimental for cardiac function in the long run. In fact, the adrenergic receptors blocker has been shown to reduce mortality in patients with heart failure^14^. Agents act by binding to adrenoceptor could directly stimulate the enzyme adenylyl cyclase (AC) converting ATP to the second messenger cAMP, which in turn binds to cAMP-dependent protein kinase A (PKA). Previous studies have demonstrated that the activity and expression of PKA will be increased during heart failure. Phosphodiesterases (PDEs), the enzymes that degrade cAMP, have an important role in regulating cAMP/PKA signaling in cardiac myocytes^15^. Additionally, the multifunctional Ca(2+)/calmodulin-dependent protein kinase II (CaMKII) is also activated in response to βAR/PKA stimulation, and is involved in the regulation of myocardial apoptosis^16^. Collectively, the previous studies suggested that long time overstimulation of βAR/cAMP-PKA-CaMKII signaling pathway could result in impairment of cardiac function.

The present study is to explore whether and why Fuzi and Beimu combination caused any adverse effects, which would be an exemplification of the research on the safety of drugs compatibility. Our previous studies have demonstrated that the alcohol amine alkaloids of Fuzi could active β_2_-AR^17^. We hypothesized that Fuzi and Beimu combination is prone to develop severe heart adverse side-effects by synergic activating of cAMP-PKA-CaMKII signaling pathway. In the present study Monocrotaline (MCT) induced rats PH model which results in compensatory right ventricular hypertrophy and heart failure^18^,^19^,^20^, were employed to evaluate the efficacy and safety of the combination treatment of Fuzi and Beimu.

## Results

### Fuzi and Beimu combination improved lung function when used at the early stage of PH

**Figure 1:**
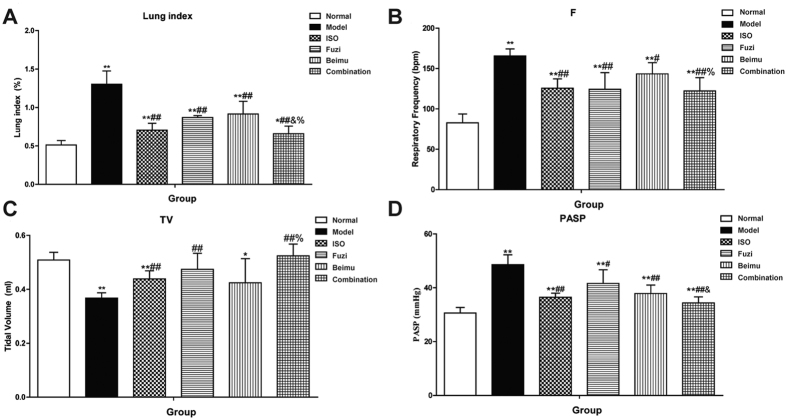
Combination of Fuzi and Beimu ameliorates lung dysfunction of the early stage of PH. Effects on lung index (**A**) pulmonary function ((**B**) respiratory frequency and (**C**) tidal volume) and pulmonary hemodynamic characteristics ((**D**) pulmonary artery systolic pressure and (**E**) pulmonary arterial diastolic pressure). Data are presented as mean ± S.D, *P < 0.05, **P < 0.01 vs Normal; ^#^P < 0.05, ^##^P < 0.01 vs Model; ^&^P < 0.05, ^&&^P < 0.01 vs Fuzi; ^%^P < 0.05, ^%%^P < 0.01 vs Beimu.

To evaluate the effectiveness of the combination of Fuzi and Beimu on PH, we first assessed the changes of lung function, including lung index, respiratory parameters (F, TV) and pulmonary artery systolic pressure (PASP) in MCT-induced PH rats after treatment (Fig. 1). Two weeks after MCT injection, model rats showed a marked increase in lung index (PASP and F), and a significant decrease in TV compared to the normal group. Combination treatment of Fuzi and Beimu resulted in a greater improvement in lung index (Fig. 1A), respiratory frequency (Fig. 1B) and pulmonary artery systolic pressure (Fig. 1D), as well as markedly increased tidal volume (Fig. 1C), compared with Fuzi or Beimu monotherapy.

**Figure 2:**
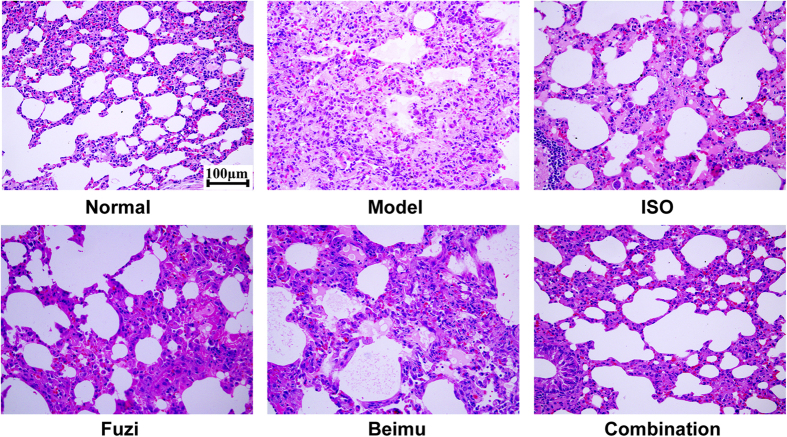
Pathology changes of the lung tissue (hematoxylin and eosin staining) at early phase of PH.

Representative micrographs of pulmonary pathological morphology were shown in Fig. 2. MCT-induced model group showed the following histological lesions: alveolar septum widening, pulmonary arteriolar wall thickening with hyaline degeneration and inflammatory cell infiltration obviously, lumens stenosis or even obliteration. However, Fuzi and Beimu combination treatment attenuated these structural abnormalities (Fig. 2). These results suggested that Fuzi and Beimu combination treatment could improve lung function at the early stage of PH.

Fuzi and Beimu combination had no significant changes on heart at early phase of PH (Supplementary Fig. S1 and S2).

### Fuzi and Beimu combination aggravated cardiotoxicity when used at the late stage of PH

**Figure 3:**
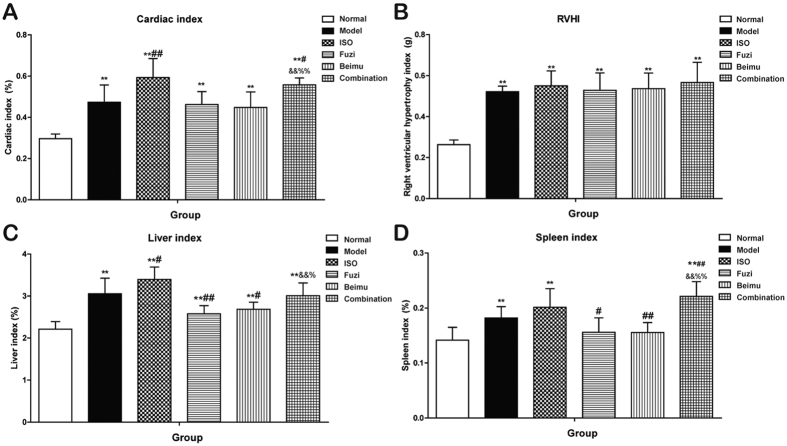
Effects of Combination on visceral index at late phase of PH. (**A**) Cardiac index. (**B**) Right ventricular hypertrophy index (RVHI). (**C**) Liver index. (**D**) Spleen index. Data are presented as mean ± S.D. *P < 0.05, **P < 0.01 vs Normal; ^#^P < 0.05, ^##^P < 0.01 vs Model; ^&^P < 0.05, ^&&^P < 0.01 vs Fuzi; ^%^P < 0.05, ^%%^P < 0.01 vs Beimu.

To evaluate whether the combination of Fuzi and Beimu could lead to more cardiotoxicity, treatment in PH rats was initiated 4 weeks after MCT injected, and the alterations of viscera index was measured after two weeks of oral administration (Fig. 3). MCT-treated rats showed significant increases in cardiac, liver and spleen index as well as right ventricular hypertrophy index (RVHI). Nevertheless, Fuzi and Beimu combination further elevated cardiac, liver and spleen index compared with each drug alone, suggesting that Fuzi and Beimu combination resulted in aggravated toxicity.

**Figure 4:**
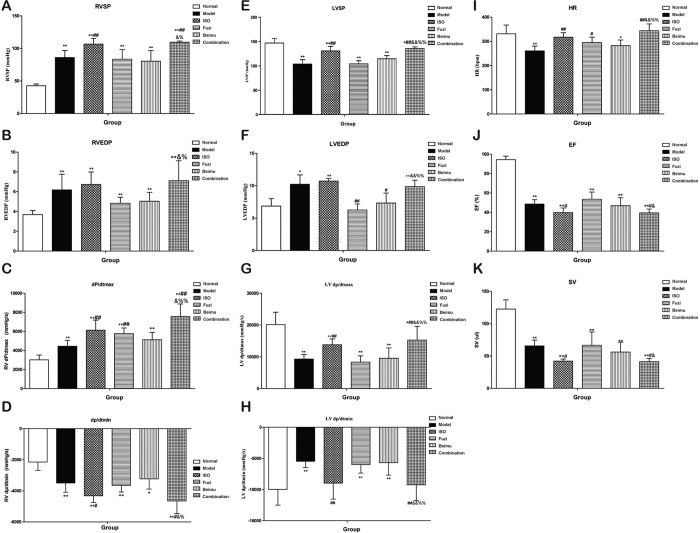
Combination aggravates heart failure of the late stage. (**A**–**D**) Hemodynamic parameters assessed for right ventricle. (**E**–**I**) Hemodynamic indexes measured in left ventricular function. (**J**,**K**) Indicators of cardiac function tested by transthoracic doppler echocardiography. Data are presented as mean ± S.D. *P < 0.05, **P < 0.01 vs Normal; ^#^P < 0.05, ^##^P < 0.01 vs Model; ^&^P < 0.05, ^&&^P < 0.01 vs Fuzi; ^%^P < 0.05, ^%%^P < 0.01 vs Beimu.

**Figure 5:**
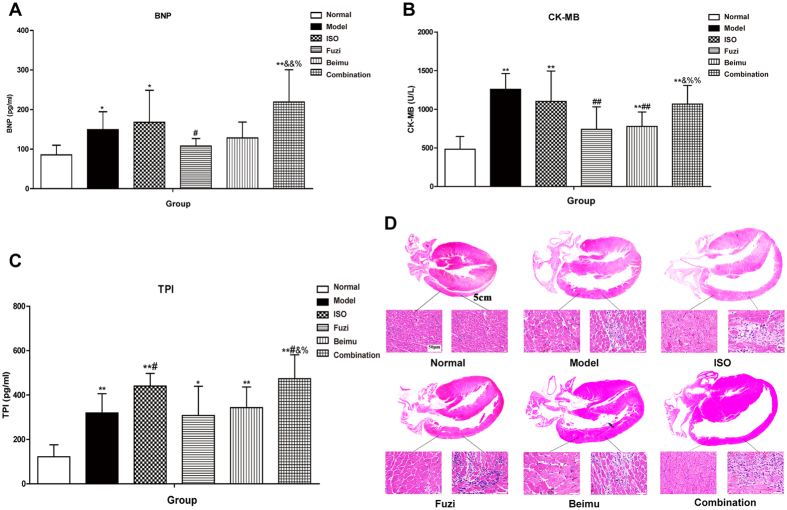
Indicators of myocardial injury of late stage of PH. Serum levels of BNP (**A**), CK-MB (**B**) and TPI (**C**). And pathology changes of cardiac tissue at the late stage of PH. Data are presented as mean ± S.D. *P < 0.05, **P < 0.01 vs Normal; ^#^P < 0.05, ^##^P < 0.01 vs Model; ^&^P < 0.05, ^&&^P < 0.01 vs Fuzi; ^%^P < 0.05, ^%%^P < 0.01 vs Beimu.

To further investigate the effects of Fuzi and Beimu combination on cardiac function in MCT-induced PH, echocardiographic and hemodynamic analysis were performed. Right ventricular and left ventricular indexes included (RVSP, RVEDP, RV-dP/dtmax and dP/dtmin; LVSP, LVEDP, LV-dP/dtmax and dP/dtmin, HR, EF, SV). Right ventricular function (Fig. 4A–D) was deteriorated as evidenced by increases in RV systolic pressure (P < 0.05), RV end-diastolic pressure (P < 0.05), dP/dtmax (P < 0.05), as well as decreased dP/dtmin (P < 0.05) of the rats treated with Fuzi and Beimu combination compared with each drug alone. Left ventricular overload (Fig. 4E–I) was compensatorily increased, as demonstrated by significant rises in LV systolic pressure, LV end-diastolic pressure, HR and LV-dP/dtmax, as well as markedly decreased dP/dtmin of the rats treated with Fuzi and Beimu combination compared with each drug alone. Ultimately, Fuzi and Beimu combined treatment also impaired left ventricular function, leading to reduction in LV ejection fraction (Fig. 4J, P < 0.05) and stroke volume (Fig. 4K, P < 0.05). Accordingly, we investigated the production of specific cardiac damage markers, such as serum BNP, CK-MB and TPI. As showed in Fig. 5A–C, the combination of Fuzi and Beimu-treated rats revealed a significant rise in serum levels of BNP, TPI and CK-MB compared with each drug alone. These results further suggested increased cardiotoxicity after the Fuzi and Beimu combination.

Histopathological findings confirmed cardiomyocyte specific suffering in Fuzi and Beimu combination-treated rats at the late stage of PH (Fig. 5D). No histological abnormalities were observed in normal group, whereas MCT-induced cardiac damage was featured by right ventricular chamber dilation, right ventricular anterior wall pulmonary conus significantly bulging, hyperplasia of fibroblasts, and inflammatory cell infiltration with local myocardial necrosis. Moreover, these pathological changes were aggravated in the rats treated with the combination of Fuzi and Beimu, characterized by further enlarged right ventricular chamber, unclear muscle fiber direction, and dissolved the myocardial cells sarcoplasm. Collectively, these results indicated that the development of PH would lead to heart dysfunction, and the combination of Fuzi and Beimu aggravated cardiotoxicity when used at the late stage of PH.

### PDK1/Akt/PDE4D axis was involved in the synergistic effect of Fuzi and Beimu combination

**Figure 6:**
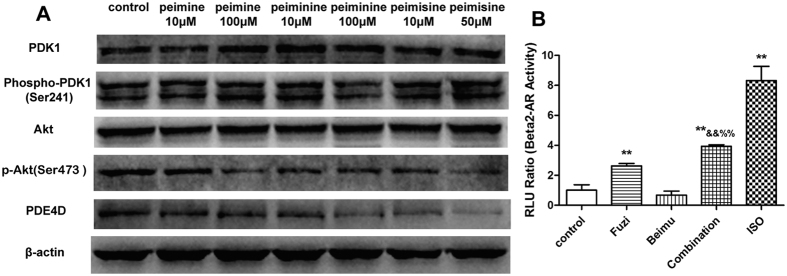
Active components of Beimu inhibit PDK1/Akt/PDE axis, down-regulating the phosphorylation of Akt and PDE. Western blot analysis of p-PDK1, PDK1, p-Akt, Akt, and PDE4D levels in H9C2 cells (**A**) n = 3. Synergistic effect of Beimu with Fuzi on increasing cAMP levels (**B**). The influence of Combination on activating β_2_AR in β2 HEK 293 cells by a Luciferase reporter gene assay (n = 6). *P < 0.0,5, **P < 0.01 vs control.

Our previous study demonstrated that alcohol amine alkaloids of Fuzi exhibited β_2_-AR stimulating effect by increasing cAMP levels in HEK 293T cells^17^. In the present study, we further evaluated whether Fuzi and Beimu combination could synergistically increase cAMP levels in β_2_AR transfected HER293 cells (Fig. 6B). The results showed that cAMP levels were significant up-regulated by the combination of Fuzi and Beimu, compared with control and two monotherapies.

To elucidate the synergistic composition in Beimu, the main chemical constituents (Peimine, Peiminine, Peimisine) of Beimu were selected. We performed a western blot assay to investigate PDK1/Akt/PDE4D axis protein expression, which signals regulated intracellular cAMP accumulation^21^. As shown in Fig. 6A, neither PDK1 nor p-PDK1 levels exhibited prominent changes following peimine, peiminine or peimisine treatment, Akt expression also displayed no apparent changes. However, the levels of p-Akt Ser473 and PDE4D were decreased after peimine, peiminine or peimisine treatment. These results indicated that PDK1/Akt/PDE4D axis is involved in the synergistic effect of Fuzi and Beimu combination treatment on activating cAMP signals.

### βAR-Gs-PKA/CaMKII signaling pathway contributed to the cardiotoxicity mediated by Fuzi and Beimu combination

**Figure 7:**
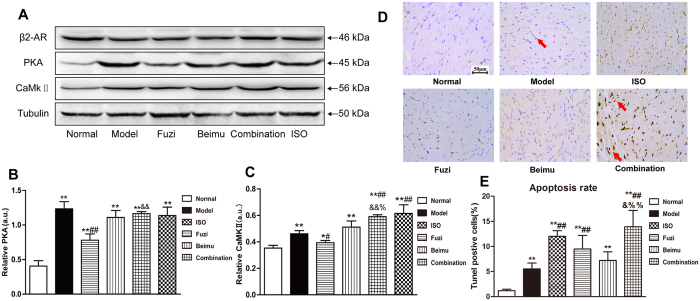
Combination up-regulates protein expressing of βAR-Gs-PKA/CaMKII singnal in right ventricles of late stage of PH (**A**–**C**). DNA fragmentation of apoptotic cells was detected by TUNEL staining in myocardium (**D**), and prcentages of apoptotic cardiomyocytes in different groups were calculated (**E**). Data are presented as mean ± S.D. *P < 0.05, **P < 0.01 vs Normal; ^#^P < 0.05, ^##^P < 0.01 vs Model; ^&^P < 0.05, ^&&^P < 0.01 vs Fuzi; ^%^P < 0.05, ^%%^P < 0.01 vs Beimu.

It has been reported that persistent βAR stimulation through Gs/PKA/CaMKII signaling induces a multitude cardiac toxicity, such as myocardial hypertrophy and apoptosis^22^,^23^,^24^. In the present study, βAR-Gs-PKA/CaMKII signaling pathway was assessed using Western blot. The results showed that PKA and CaMKII protein levels were significantly up-regulated in MCT-treated rats compared to untreated control (Fig. 7A–C). Interestingly, rat treated with Fuzi and Beimu combination further up-regulated PKA and CaMKII proteins compared with the two monotherapies in heart tissues. These results suggested that activation of PKA/CaMKII signaling pathways may induce myocardial apoptosis at the late stage of PH. As expected, MCT-treated rats showed significant induction of cardiomyocyte apoptosis, whereas apoptosis was notably further induced in myocardial sections of Fuzi and Beimu combination group, as demonstrated by TUNEL immunostaining (Fig. 7D,E).

**Figure 8:**
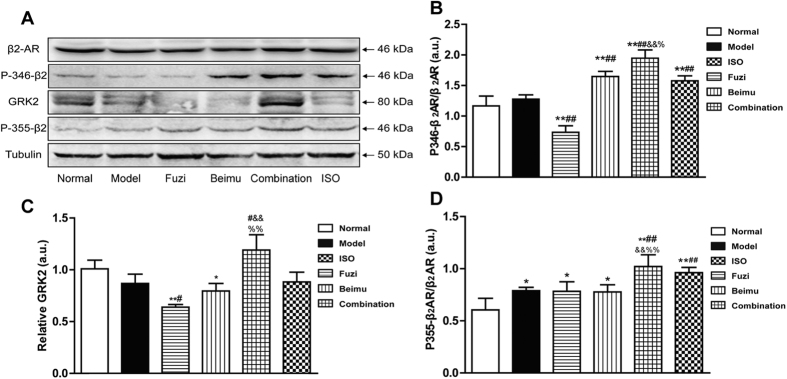
Combination up-regulates protein expressing of PKA/GRK2-β_2_AR-Gi pathways in right ventricles of late stage of PH (**A**–**D**).

Previous studies have suggested that phosphorylation of β_2_AR by PKA induces the switching of the coupling receptor from Gs to Gi^25^,^26^. Moreover, GRK2-dependent β_2_AR-Gi signaling in cardiac tissue is a primary negative regulator of βAR pro-contractile signaling, which contributes to the pathogenesis and progression of heart failure^27^,^28^,^29^. Ser(355,356) sites were associated with GRK phosphorylation of the β_2_AR, and Ser346 was PKA consensus site^30^,^31^. Thus, we examined GRK2, P346-β_2_AR and P355-β_2_AR protein expression. As shown in Fig. 8, protein levels of GRK2, P346-β_2_AR and P355-β_2_AR were largely increased after Fuzi and Beimu combination treatment. The results indicated that deterioration of heart failure during Fuzi and Beimu combination treatment was associated with the activation of βAR-Gs-PKA/CaMKII signaling pathway.

## Discussion

The present study for the first time identified an additive heart adverse effect resulting from a typical TCM incompatibility (Fuzi and Beimu) in treating PH. Our results showed that when administrated at the early stage of PH, the combination of Fuzi and Beimu showed a prominent therapeutic efficacy, characterized by decreasing lung weight, improving lung function (respiration frequency and tidal volume), as well as abrogating the development of pulmonary arterial hypertension and pulmonary vascular remodeling, and the combination treatment had no serious effect on heart and liver (Supplementary Figs S1 and S2) in this stage. However, when used at the late stage of PH, it led to serious heart adverse effects, evidenced by exacerbating right ventricular failure (cardiac index and right ventricular pressure), as well as compensated left ventricular hyperfunction and eventually heart failure (heart rate, left ventricular pressure, ejection fraction, stroke volume and apoptosis). The increased risk of heart adverse effects may be attributed to the synergistic activation of cAMP-PKA- CaMKII signal pathway.

A recent study demonstrated that higenamine in Fuzi or Chuanwu was a β_2_AR agonist, which resulted in muscular relaxation. Our previous study reported that hypaconine, aconine, chasmanine and karakolidine in Fuzi or Chuanwu were β2-AR agonists^17^,^21^. We therefore hypothesized that Fuzi and Beimu combination could have the potential to synergistically improve lung dysfunction. However, prolonged activation of β_2_AR has been shown to lead to cardiac dysfunction and eventually development of dilated cardiomyopathy^25^,^32^. Therefore, Fuzi and Beimu combination would be likely to aggravate heart failure at the late state of PH. Consistent with previous reports, our data showed that MCT injection remarkably increased F and reduced TV compared to untreated animals, while the Fuzi and Beimu combination treatment at the early stage of PH notably decreased F, and meanwhile markedly elevated TV. A significant elevation of lung index such as the hyperplasia of pneumonocyte and presence of an extensive proliferative pulmonary was observed in MCT-induced PH rats^33^, whereas this elevation was mitigated by the combination of Fuzi and Beimu. Taken together, these results strongly supported the beneficial effect of Fuzi and Beimu combination in pulmonary hypertension.

RV hypertrophy and failure in MCT-treated rats were manifested with a significant elevation in heart/body weight and RV/LVS ratios, hemodynamically by an increase in values of RVEDP, RVSP, and RV dP/dtmax and RV dP/dtmin, and histopathologically by the evidence of cardiotoxicity. The congested liver and spleen in the late stage of PH further confirmed the existence of the RVF^34^,^35^. Our results suggested that the Fuzi and Beimu combination exacerbated these dysfunctional parameters of RV, leading to RV dilatation and wall thinning when used at the late stage of PH. Rats with MCT injection developed LV systolic and diastolic dysfunction, as defined by decreased LVSP and dP/dtmax, as well as elevated LVEDP and dP/dtmin. Interestingly, Fuzi and Beimu combination treatment increased LV function, further validated by elevation in values of LVSP, LVEDP, LV-dP/dtmax and dP/dtmin, and HR. Overall, these sequential events triggered hyperfunctions, and a vicious cycle would ensue the increased energy consumption and cardiac maladaptive remodeling. Echocardiographic outcomes further suggested cardiac systolic function was impaired as evidenced by a decrease in LV ejection fraction and stroke volume. TUNEL staining showed that apoptosis was strikingly more apparent in myocardial sections of Fuzi and Beimu group. In addition, BNP, TPI and CK-MB levels, which have been suggested as biomarkers for cardiac dysfunction, were further elevated in PH rats treated by Fuzi and Beimu combination^36^,^37^. In summary, these results suggested that Fuzi and Beimu combination aggravated heart failure.

**Figure 9:**
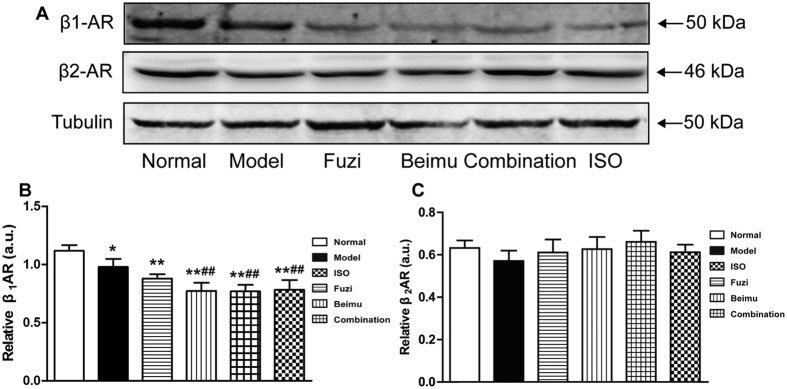
Effects of incompatibility on β-AR in right ventricles, protein levels of β_1_AR and β_2_AR were analyzed by Western blotting (n = 3). Data are presented as mean ± S.D. *P < 0.05, **P < 0.01 vs Normal; ^#^P < 0.05, ^##^P < 0.01 vs Model; ^&^P < 0.05, ^&&^P < 0.01 vs Fuzi; ^%^P < 0.05, ^%%^P < 0.01 vs Beimu.

Previous studies have suggested that β_1_AR was selectively down-regulated in human HF, and thus shifted the stoichiometry of β_1_AR:β_2_AR from ~75:~20 in the normal and healthy heart towards 50:50 in the heart failure, resulting in a relative increase in the proportion of cardiac β_2_AR^26^,^38^. Therefore, we focused on the β_2_AR, in particular the Gs/Gi signaling pathway. Consistently, we observed β_1_AR was markedly down-regulated in heart failure, whereas the expression of β_2_AR showed no obvious changes (Fig. 9). Recent study indicated that β_2_AR activation increased the incidence of ventricular arrhythmia in the experimental heart failure model^39^. To explore the effect of Fuzi and Beimu combination on β_2_AR activation, a transfected HEK293 cells over-expressing β_2_AR was employed. Our results showed that Beimu had a synergistic effect with Fuzi on increasing cAMP levels (Fig. 6B). Besides, we found some chemical components of Beimu down-regulated both Akt phosphorylation and PDE levels (Fig. 6A). Therefore, the synergistic β_2_AR stimulation may be resulted from PDK1/Akt/PDE4D axis inhibition. Both β-AR subtypes were able to activate the classic Gs-adenylyl cyclase-cAMP-protein kinase A (PKA) signaling pathway, which elicited inotropic and chronotropic effects^40^. In the heart failure, persistent β-AR stimulation has been shown through PKA-independent and PKA-dependent activation of calcium/calmodulin-dependent kinase II (CaMKII), which has been shown to activate apoptotic signaling pathways. Moreover, overwhelming evidence indicated increase in cardiac CaMKII activity promotes cardiomyocyte apoptosis and cardiac remodeling^22^,^32^,^41^. However, our study revealed that the protein levels of PKA and CaMKII in Fuzi and Beimu combination group were significantly increased, suggesting that the exaggerated pathological consequences were associated with the activation of βAR-Gs-PKA/CaMKII signaling pathway.

Compelling evidence has shown that upregulation of GRK2 by exaggerated β_2_AR-Gi signaling was a causal factor in maladaptive cardiac remodeling and the progression of HF. Several animal studies have shown that expression of a peptide inhibitor of GRK2 (βARKct) can ameliorate the systolic function, including promoting reverse remodeling of the LV. Moreover, β2AR phosphorylation mediated by PKA was a prerequisite for β_2_AR-Gi coupling^27^,^28^,^29^,^42^. The present study demonstrated that protein levels of GRK2, P346-β_2_AR and P355-β_2_AR were markedly increased after Fuzi and Beimu combination. Therefore, the PKA/GRK2-β_2_AR-Gi signaling transduction pathway appeared to participate in the process of cardiac function deterioration.

In summary, our present study for the first time discovered Fuzi and Beimu combination therapy could improve lung function and reduce pulmonary histopathological changes in the early stage of PH, whereas it potentially aggravated the heart injury in the late stage of PH due to the inhibition of PDK1/Akt/PDE4D axis and subsequent synergistic activation of βAR-Gs-PKA/CaMKII signaling pathway. To some extent, the present study was helpful to understand the taboo conditions of drug combination in clinical practice based on the conventional wisdom from ancient TCM knowledge, at least it could provide some research ideas, in which different symptom conditions would be the key terms leading to toxicity or efficacy of the co-use of the drugs.

## Materials and Methods

### Drug Preparation

*Aconiti Lateralis Radix Praeparata* (Fuzi) and *Fritillariae Thunbergii bulbus* (Beimu) were purchased from Chinese herbs Corporation. Fuzi (Hei Shuenpian) and Beimu (Zhe Beimu) as well as the combination (1:1) of the two herbs were crushed into coarse powder, soaked with distilled water 10 times for 0.5 h, and microboiled twice for 1 h. The merged filtrate was dried under vacuum at 60 °C, and the final powder was either stored at −20 °C or diluted with distilled water to the appropriate concentration for further application.

### Animal Model and Experiment Design

Male Wistar rats (7 weeks of age and weighing 180 to 200 g. Certificate No SCXX 2009-0004) were obtained from Vital River Laboratory Animal Technology Co., Ltd (Beijing, China). Rats were housed in an environmentally controlled breeding room (temperature: 22 ± 2 °C, humidity: 60 ± 5%, 12 h dark/light cycles). Water and food were given ad libitum. The animal care was performed strictly according to the Care and Use of Laboratory Animals of Institutional Animal Care and all experiments were approved by the Animal Ethics Committee of Tianjin University of Traditional Chinese Medicine (No.TCM-2013-LACE201312-05).

**Figure 10:**
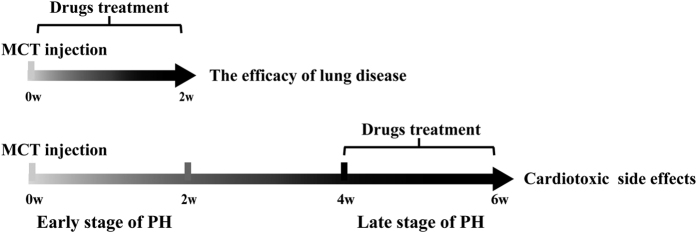
Protocols for drug administration. Wistar rats received a single intraperitoneal injected of MCT, at the early or late stage of PH, rats were administrated each drug for 2 weeks, these rats were randomly grouped, n = 20.

PH rat model was induced by a single intraperitoneal injection of monocrotaline (MCT, 60 mg/kg b.wt. GR-132-140225, GuangRun Bio Technology, Co. Ltd. Nanjing, China) previously dissolved in 1 N HCl and pH adjusted to 7.4 using 1 N NaOH, eventually causing heart failure^43^,^44^,^45^. For comparison purposes, normal control rats received a single intraperitoneal injection of saline. Based on our previous study (Data not shown), the development of MCT-induced rats PH rat mode was divided into two stages (Fig. 10). Namely drug early intervention stage (DEIS) in which each drug were administrated at the first 2 weeks after MCT injection, and drug late intervention stage (DLIS) in which each drug were administrated 4 weeks after MCT injection. Each drug was administrated for 2 weeks. After 7 days adaptive feeding, rats were divided into six groups randomly for each stage: normal control group (Normal, n = 20), model group (Model, n = 20), isoproterenol group (ISO, n = 20, ip. 0.09 mg/kg/d), Fuzi group (Fuzi, n = 20, ig. 5 g/Kg/d), Beimu group (Beimu, n = 20, ig. 5 g/Kg/d) and combination group (Combination (1:1), n = 20, ig. 5:5 g/Kg/d), 4 fold of clinical equivalent dose of the two herbs and 1:1 ratio for combination were selected based on our preliminary experiment in the animal experiment.

### Lung Function Measurement

The rats undergoing non-invasive pulmonary function were monitored by whole-body barometric plethysmography (WBP; EMKA Technologies, Paris, France)^46^. Rats were placed in a plethysmograph chamber, and a 10-min accommodation was allowed before analysis. Respiratory parameters were recorded while the rats were unrestrained, and the respiratory frequency (F) and tidal volume (TV) were analyzed by emka Technologies iOX2 software.

### Echocardiography and Hemodynamic Measurements

Rats were anesthetized with urethane (0.6 ml/100 g), and the thoraxes of the rats were shaved. Transthoracic two-dimensionally guided M-mode echocardiography was performed every week after the rats were administered, using an Ultra-high resolution small animal ultrasound scanner Vevo^®^2100 (Visual Sonics, Canada)^47^. LVIDd, LVIDs, IVSd, IVSs, LVPWd, LVPWs, LVEDV, LVESV were recorded. CUBED formula was applied to the calculation of the left ventricular ejection fraction (EF, %) and stroke volume (SV, μL) using the following equations: EF = (LVEDV − LVESV)/LVEDV×100%; SV = LVEDV − LVESV.

Hemodynamic assessments were performed using a polygraph system (Power Lab; AD Instruments, Bella Vista, NSW, Australia) at a sampling rate of 100 Hz^48^,^49^. RVSP, RVEDP, RV ± dp/dtmax, PASP, PADP, HR, LVSP, LVEDP and LV ± dp/dtmax were measured. All these data were fed to a computer using an amplitude discriminator, analyzed by Chart 7.3.7 software. The animals were sacrificed for further analysis at the end of the hemodynamic measurements.

### Tissue Processing and Histopathological Analysis

At the end of the study, the viscera (Lung, heart, liver) tissues were rapidly removed and weighed. The viscerosomatic index was calculated using the viscera weight relative to the body weight, viscerosomatic index (VSI) = 100 × (viscera weight, g)/(body weight, g). The hearts were excised with the right ventricle (RV) and left ventricle plus intraventricular septum (LVS) were weighted to calculate the RV/LVS weight ratio as a marker for right ventricular hypertrophy index (RVHI)^50^. The right ventricle of the heart was trimmed away from the left ventricle and atria and stored at −80 °C for protein quantification.

The myocardial and pulmonary tissues were fixed in 10% neutral buffered formalin and paraffin-embedded, sectioned into 5-micrometer thick, and stained with hematoxylin and eosin (H&E).

Apoptosis in cardiomyocytes was evaluated with enzymatic labeling of DNA strand breaks detected using terminal deoxynucleotidyl transferase-mediated deoxyuridine triphosphate nick end-labeling (TUNEL) stain. TUNEL staining was performed with *In Situ* Cell Death Detection kit (Roche, Mannheim, Germany) according to the manufacturer’s instructions. Images were obtained with using OLYMPUS U-CMAD3 optical microscope (OLYMPUS, Japan) pictures were taken with OLYMPUS C5060-ADU camera (OLYMPUS, Japan). The apoptotic ratio was shown as the percentage of positive staining

### Serum ELISA and Biochemical Measurements

Blood was collected, and centrifuged at 3000 rpm at 4 °C for 15 minutes to separate serum. CK-MB was tested by the semi-automatic biochemical analyzer. The levels of TPI, BNP, ET-1 and NO in serum were determined by ELISA kit (Shanghai BlueGene Biotech CO., LTD., Shanghai, China) in accordance with the manufacture’s protocols.

### Cell Culture and Luciferase Reporter Assay for β2AR Activation

The rat embryonic cardiomyoblastic cell line H9C2 was cultured in Dulbecco’s modified Eagle’s medium (DMEM, HyClone, USA) supplemented with 10% FBS and 1% penicillin-streptomycin (HyClone, USA) in a 37 °C, 5% CO_2_ incubator. Cell medium was changed every 2 days. H9C2 cells were plated on the appropriate culture plates and treated with peimine (10 μmol/L, 100 μmol/L), peiminine (10 μmol/L, 100 μmol/L) or peimisine (10 μmol/L, 50 μmol/L) for 24 h, and then the protein of cells was extracted for western blot analysis. HEK293 cell line over-expressing β2-adrenergic receptor was cultured in DMEM supplemented with 10% fetal bovine serum (FBS, Biological industries), 100 U/ml penicillin and streptomycin, 100 ug/ml 0.25% Zeocin (Invitrogen Corporation, Carlsbad, CA, USA) at 37 °C in a 95% air and 5% CO_2_ humidified atmosphere. Cells were cultured in 75 cm^2^ culture flakes, and plated on the 96 well cell culture cluster. Once they reached 30–40% confluence, cells were co-transfected with solution A: luciferase plasmid pGL4.29 (Promega Corporation, USA) and pRL-TK vector, and solution B: transfection reagents PEI (Invitrogen, Corporation, Carlsbad, CA, USA), After overnight incubation, the cells were washed with PBS and incubated with fresh medium for 24 h, prior to the addition of Fuzi, Beimu, Fuzi and Beimu combination, as well as positive control Isoproterenol for 5 h. The luciferase activity was measured by Dual-Glo^TM^ reporter assay system (Promega Corporation, USA) according to the manufacture’s protocol. The firefly/Renilla luciferase activity ratio was used to compare the differences in transfection efficiency. Results are presented as mean ± S.D. of three independent experiments performed in duplicate.

### Western blot Analysis

Western blot assay was performed as follows. Briefly, the protein of the H9C2 cells or cardiac tissues was extracted using a Total Protein and Neuclear-Cytosol Extraction Kit (Beyotime Institute of Biotechnology Inc., shanghai, China) following the manufacturer’s protocols. BCA assay was used to measure the protein concentration. Separated by sodium-dodecyl-sulfate polyacrylamide gel electrophoresis, and then transferred onto polyvinylidene fluoride membranes. Membranes were blocked with 5% skimmed milk, and then were incubated with primary antibodies Rabbit anti-tubulin beta antibody (Bioss, China) or β-actin (Bioss); PDE4D (Santa Cruz, USA); PDK1, p-PDK1(Ser241) Akt, p-Akt (Ser473) (CST, USA); anti-beta 1 adrenergic receptor antibody, anti-beta 2 adrenergic receptor antibody, anti-GRK2 antibody (Abcam); anti-PKA antibody (millipore); anti-CaMK2antibody(CST); β2-AR phosphorylated serine pSer346 and pSer(355,356) (sigma), overnight at 4 °C. Secondary antibody goat anti-rabbit antibody (ZSGB-BIO ORIGENE, BEIJING, China) were diluted to identify the corresponding primary antibodies. Further analysis was carried out by using an imaging densitometer (Cuene Genins) to quantify the immunoreactive bands.

### Statistical Analysis

The SPSS software (IBM SPSS Statistics 20.0) was used for statistical analysis. Quantitative data were presented as mean ± SD., the differences among various groups were performed by one-way analysis of variance (ANOVA), non-parametric equivalents were performed as appropriate. Enumeration data were compared with Chi-square Test. P < 0.05 was considered statistically significant.

## Additional Information

**How to cite this article**: Zhuang, P. *et al*. cAMP-PKA-CaMKII signaling pathway is involved in aggravated cardiotoxicity during Fuzi and Beimu Combination Treatment of Experimental Pulmonary Hypertension. *Sci. Rep.* **6**, 34903; doi: 10.1038/srep34903 (2016).

## Supplementary Material

Supplementary Information
