# Supplementary material for: cAMP-PKA-CaMKII signaling pathway is involved in aggravated cardiotoxicity during Fuzi and Beimu Combination Treatment of Experimental Pulmonary Hypertension
Source: Sci Rep. 2016 Oct 14;6:34903. doi: 10.1038/srep34903 (PMC5064387; doi:10.1038/srep34903)
Supplement: Supplementary Information [file srep34903-s1.doc]

**cAMP-PKA-CaMKII signaling pathway is involved in aggravated cardiotoxicity during Fuzi and Beimu Combination Treatment of Experimental Pulmonary Hypertension**

Pengwei Zhuang, Yingying Huang, Zhiqiang Lu, Zhen Yang, Liman Xu, Fengjiao Sun, Yanjun Zhang, Jinao Duan

**Supplementary Information**

**
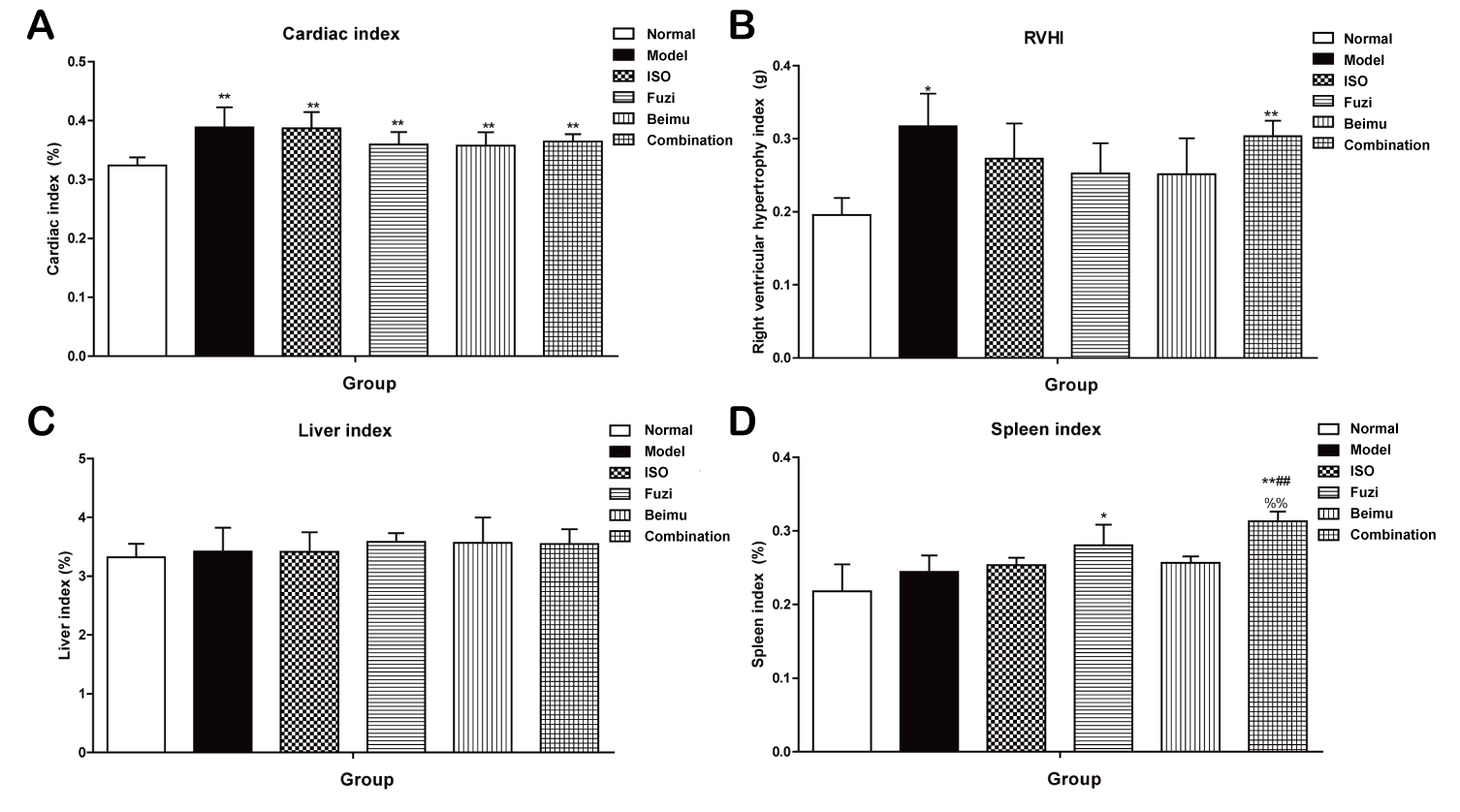
**

Supplementary Fig.S1 Effects of Combination on visceral index at early phase of PH. (A) Cardiac index. (B) Right ventricular hypertrophy index (RVHI). (C) Liver index. (D) Spleen index. Data are presented as mean ±S.D. *P<0.05, **P<0.01 vs Normal; #P<0.05, ##P<0.01 vs Model; &P<0.05, &&P<0.01 vs Fuzi; %P<0.05, %%P<0.01 vs Beimu.


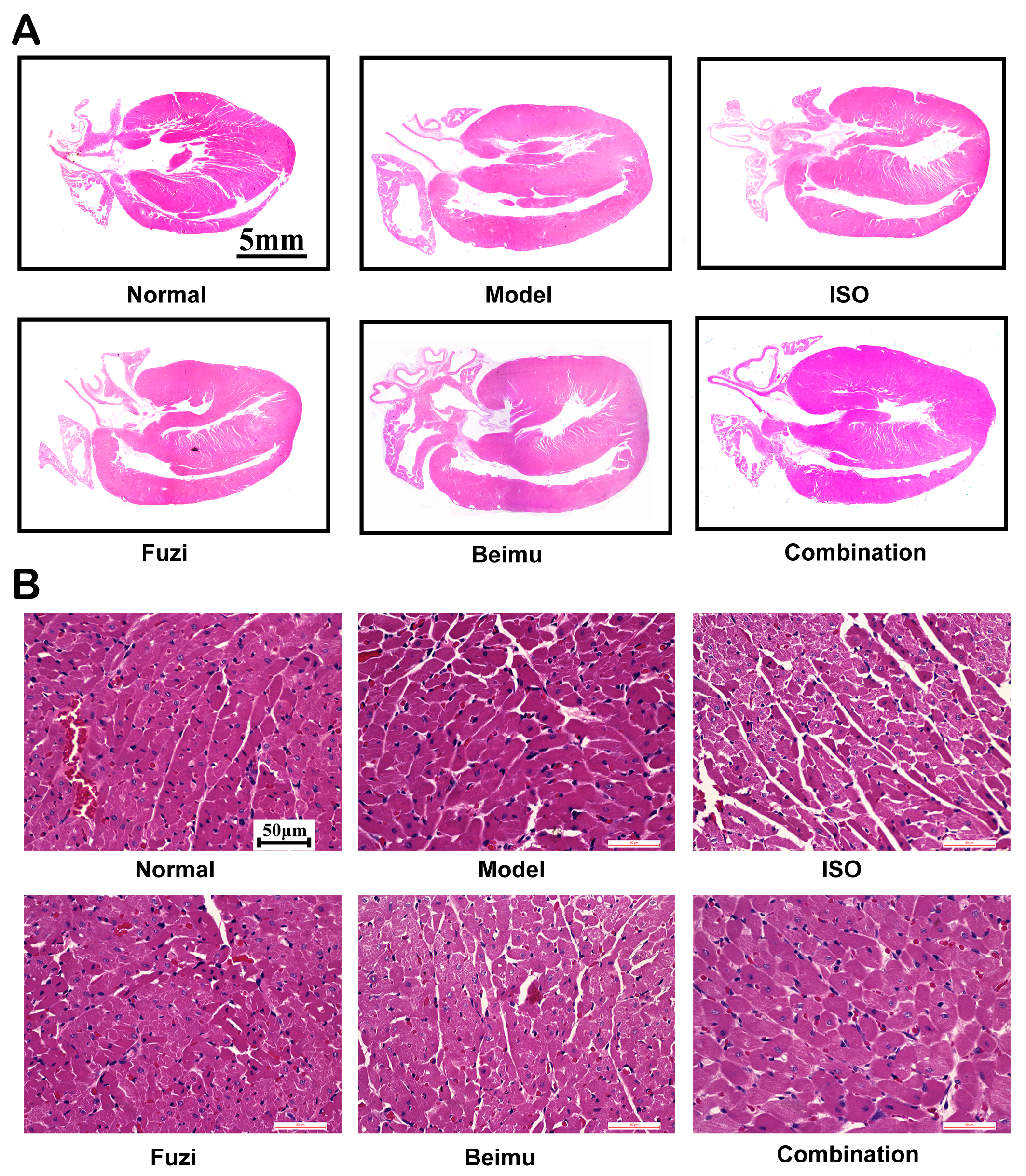


Supplementary Fig.S2. Combination treatment has no serious effect on cardiac histopathology at early phase of PH.
